# Fluid resuscitation therapy in endotoxemic hamsters improves survival and attenuates capillary perfusion deficits and inflammatory responses by a mechanism related to nitric oxide

**DOI:** 10.1186/s12967-014-0232-z

**Published:** 2014-08-24

**Authors:** Nivaldo Ribeiro Villela, Ana Olimpia Maia Teixeira dos Santos, Marcos Lopes de Miranda, Eliete Bouskela

**Affiliations:** Department of Surgery, Division of Anesthesiology, Faculty of Medical Sciences, Rio de Janeiro State University, Boulevard 28 de Setembro, 77 - Vila Isabel, 20.551-030 Rio de Janeiro, RJ Brazil; Oswaldo Cruz Foundation - Fiocruz, Main Campus, Av. Brazil 4365, Manguinhos, 21040-360 Rio de Janeiro, RJ Brazil; Laboratory for Clinical and Experimental Research in Vascular Biology - BioVasc, Pavilhão Reitor Haroldo Lisboa da Cunha, Rio de Janeiro State University, Rua São Francisco Xavier 524, 20550-013 Rio de Janeiro, RJ Brazil

**Keywords:** Sepsis, Endotoxemia, Nitric oxide, Fluid resuscitation, Microcirculation

## Abstract

**Background:**

Relative hypovolemia is frequently found in early stages of severe sepsis and septic shock and prompt and aggressive fluid therapy has become standard of care improving tissue perfusion and patient outcome. This paper investigates the role of the nitric oxide pathway on beneficial microcirculatory effects of fluid resuscitation.

**Methods:**

After skinfold chamber implantation procedures and endotoxemia induction by intravenous *Escherichia coli* lipopolysaccharide administration (2 mg.kg^−1^), male golden Syrian hamsters were fluid resuscitated and then sequentially treated with L-N^*ω*^-Nitroarginine and L-Arginine hydrochloride (LPS/FR/LNNA group). Intravital microscopy of skinfold chamber preparations allowed quantitative analysis of microvascular variables including venular leukocyte rolling and adhesion. Macro-hemodynamic, biochemical and hematological parameters as well as survival rate were also evaluated. Endotoxemic hamsters treated with fluid therapy alone (LPS/FR group) and non-treated animals (LPS group) served as controls.

**Results:**

Fluid resuscitation was effective in reducing lipopolysaccharide-induced microcirculatory changes. After 3 hours of lipopolysaccharide administration, non-fluid resuscitated animals (LPS group) had the lowest functional capillary density (1% from baseline for LPS group *vs.* 19% for LPS/FR one; *p* <0.05). At the same time point, arteriolar mean internal diameter was significantly wider in LPS/FR group than in LPS one (100% *vs.* 50% from baseline). Fluid resuscitation also reduced leukocyte-endothelium interactions and sequestration (*p* <0.05 for LPS *vs.* LPS/FR group) and increased survival (median survival time: 2 and 5.5 days for LPS and LPS/FR groups, respectively; *p* <0.05). Nitric oxide synthase inhibition prevented these protective effects, while L-Arginine administration markedly restored many of them.

**Conclusion:**

Our results suggest that the underlying mechanism of fluid therapy is the restoration of nitric oxide bioavailability, because inhibition of NOS prevented many of its beneficial effects. Nevertheless, further investigations are required in experimental models closer to conditions of human sepsis to confirm these results.

## Background

Sepsis is an infection-related systemic inflammatory syndrome with high incidence, morbidity, mortality, and cost to the healthcare system [1,2]. As relative hypovolemia is frequently found in early stages of severe sepsis and septic shock, prompt and aggressive fluid therapy has become standard of care. The so-called fluid resuscitation can restore intravascular volume improving macro-hemodynamic parameters, tissue perfusion, and even patient outcome [3-6]. Despite these well-established beneficial effects of fluid resuscitation, some authors recently observed an increased mortality related to aggressive fluid therapy in septic patients [7-9].

These contradictory results address an important clinical question: which population would or would not benefit from fluid therapy? This is an open field for research and discussion and probably has multiple answers some of which involving the understanding of the mechanisms behind the beneficial and detrimental effects of fluid resuscitation. Although several groups, including our own, have been studying the role of fluid resuscitation during sepsis, these mechanisms are not completely elucidated even though it is argued that they could be related to endothelium via the nitric oxide (NO) pathway [10-12].

NO is involved in a wide variety of crucial regulatory and signaling mechanisms. In spite of its physiological beneficial actions, its overproduction is linked to pathogenesis of numerous acute and chronic inflammatory processes such as septic shock hypotension. In fact, dysfunction of the endothelial NO pathway is a landmark of sepsis syndrome [13].

Thus, the present experimental study was carried out to investigate the role of NO on microcirculatory changes induced by fluid resuscitation in endotoxemic hamsters.

## Methods

Experiments were performed on 30 male golden Syrian hamsters (*Mesocricetus auratus,* ANILAB, Animais de Laboratório, Paulínea, SP, Brazil) with free access to water and standard chow (NUVILAB CR1, Quimtia S/A, Colombo, PR, Brazil). Animals were housed, one per cage, under controlled conditions of light (12:12 hours light/dark cycle) and temperature (21.0 ± 1.0°C). All procedures were approved by Rio de Janeiro State University Animal Care and Use Committee (protocol number CEUA/060/2010) and are consistent with the United States National Institutes of Health Guide for the Care and Use of Laboratory Animals (National Research Council, 1996).

### Animal preparation

The dorsal window chamber implantation procedure has been described previously by Endrich and co-workers [14] in details. Briefly, under anesthesia with sodium pentobarbital (90 mg.kg^−1^ intraperitoneal injection; Hypnol 3%, Syntec, Cotia, SP, Brazil), animals’ dorsal hair was shaved and depilated with commercial hair-removing solution. After that, the dorsal skin of the back was lifted away from the animal, creating a skinfold that was sandwiched between two titanium frames after one of its layers was microsurgically excised (circular area of 15 mm in diameter). The remaining layer, consisting of epidermis, subcutaneous tissue, and thin striated skin muscle (panniculus carnosus muscle) was covered with a removable circular cover glass incorporated into one of the metal frames, creating the window chamber. After a recovery period of 6 days, animals were reanesthetized and the left carotid artery was catheterized (polyethylene-50 catheter) allowing continuous hemodynamic monitoring and blood sampling. The left jugular vein was also catheterized (polyethylene-10 catheter) for fluid infusion and drug injection. These catheters were tunneled under the skin, exteriorized at the dorsal side of the neck, filled with heparinized saline solution (40 IU.ml^−1^), and attached to the chamber frame with tape. Experiments were performed on awake animals after 24 hours of catheter implantation.

### Hemodynamic monitoring

Mean arterial blood pressure (MAP) was continuously monitored during the experimental period through the arterial catheter connected to a pressure transducer. Analog pressure signals were digitized (MP100 Data Acquisition System, BIOPAC Systems, Goleta, CA, USA) and processed using data acquisition software for hemodynamic experiments (AcqKnowledge Software v. 3.5.7, BIOPAC Systems, Goleta, CA, USA). Heart rate (HR) was determined from the pressure trace and expressed as beats per minute (bpm).

### Intravital microscopy

The unanesthetized animal was placed in a restraining plexiglass tube attached to the stage of an intravital microscope (Ortholux, Leitz, Wetzlar, Germany) equipped with an epifluorescence assembly (100-W HBO mercury lamp with filter blocks, Leitz, Wetzlar, Germany). The body temperature of the hamsters was maintained with a heating pad placed near the animal and controlled by a rectal thermistor (LB750, Uppsala Processdata AB, Uppsala, Sweden). Moving images of the microcirculation were obtained using a 20x objective (CF SLWD Plan EPI 20x/0.35 Achromat Objective WD 20.5 mm, Nikon, Tokyo, Japan) and a charge-coupled device digital video camera system (SBC-320P B/W Camera, Samsung, Seoul, South Korea), resulting in a total magnification of 800-fold at the video monitor. Microcirculatory acquired images were recorded as video files in digital media for later evaluation. Quantitative off-line analysis of the videos was performed using Cap-Image 7.2, a computer-assisted image analysis system (Dr.Zeintl Biomedical Engineering, Heidelberg, Germany [15]), by an investigator blinded to the drug treatment. In each animal, 3 arterioles, 3 venules, and 10 capillary fields were chosen taking into account the absence of inflammation or bleeding in the microscopic field and the presence of histological landmarks that could facilitate subsequent return to the same field, since the same vessels and capillary fields were studied throughout the experiment. Arteriolar and venular mean internal diameters were measured as the perpendicular distance (in micrometers) between the vessel walls. Arteriolar blood flow velocity was determined by semiquantitative score using an ordinal scale [16]: 0, no flow; 1, intermittent flow; 2, sluggish flow; 3, normal flow. The functional capillary density (FCD) was considered to be the total length (in centimeters) of spontaneously red blood cell (RBC)-perfused capillaries per square centimeter of tissue surface area (cm.cm^−2^). RBC velocity in capillaries (RBC-Vel) was assessed by frame-to-frame analysis and determined as the ratio between the capillary distance traveled by an erythrocyte and the time required for this displacement (expressed as mm.s^−1^). One capillary per capillary field was studied in each animal during RBC-Vel assessment. The selection of this capillary was based on two criteria: it should be representative of the mean RBC-Vel of that field and have good image quality for reliable analysis.

### Evaluation of leukocyte-endothelial interactions

After *in vivo* staining of leukocytes with rhodamine 6G (0.15 mg.kg^−1^ intravenously [IV]; 0.4 ml; Sigma-Aldrich, St. Louis, MO, USA), leukocyte-endothelial interactions were assessed by intravital fluorescence microscopy. According to their interaction with the microvascular endothelium, leukocytes were classified as passing, rolling, or adhered. Passing leukocytes were defined as white blood cells traversing an observed venular segment without sticking contact (adherence) to the endothelium lining and rolling leukocytes as white blood cells moving along the endothelial lining at a velocity slower than that of the surrounding erythrocytes. The number of rolling leukocytes was expressed as percentage of the number of passing ones. A leukocyte was considered to be adherent to the venular endothelium lining if it remained stationary for more than 30 seconds. Adherent cells were counted in a 100 μm venular segment and the number of adherent leukocytes was expressed as the number of adherent cells per field. Cell counting was performed off-line by an investigator blinded to treatment. Three venules were studied in each animal, and a single period of 60 seconds was analyzed per venule for all cell counts.

### Biochemical and hematological parameters

Blood samples were withdrawn from the arterial catheter and immediately analyzed in a point-of-care lactate and blood gas analyzer (i-STAT System/CG4+ cartridge, Abbott Laboratories, Abbott Park, IL, USA) for arterial lactate concentrations, pH, bicarbonate level (HCO_3_), and base excess (BE). Arterial blood glucose was also immediately measured with a glucometer (OneTouch Ultra, Lifescan, Johnson & Johnson, Milpitas, CA, USA). Hematocrit was determined by the microhematocrit method using arterial blood samples collected into heparinized capillary tubes. Arterial blood leukocytes were manually counted by standard laboratory method. Briefly, after leukocyte staining with gentian violet and erythrocyte hemolysis by acetic acid (Türk’s solution, Sigma-Aldrich, St. Louis, MO, USA), leukocytes were counted in a defined volume using a Neubauer counting chamber and an optical microscope equipped with a 10x/0.25 objective (BIOVIDEO, BEL Photonics, Monza, Italy). Leukometry was expressed as the number of leukocytes per mm^3^.

### Experimental protocol

Animals were suitable for experiments if their baseline hemodynamic variables were within the normal range and if they did not have signs of inflammation and/or bleeding in the chamber.

At the beginning of the experiment, animals were given 30 minutes to adapt to the restraining plexiglass tube before baseline variables were measured. Immediately after baseline determination of hemodynamic and microcirculatory parameters and evaluation of leukocyte-endothelial interactions, endotoxemia was induced by an IV injection of 1 mg.kg^−1^ of *Escherichia coli* serotype 055:B5 lipopolysaccharide (LPS; Sigma-Aldrich, St. Louis, MO, USA) diluted in normal saline (total volume of 0.2 ml). After endotoxemia induction, animals were randomly allocated in one of three study groups: (1) LPS (n = 10) – no further treatment after LPS injection; (2) LPS/FR (n = 10) – one hour after LPS injection animals were fluid resuscitated with normal saline (40 ml.kg^−1^ in 30 minutes); (3) LPS/FR/LNNA (n = 10) – one hour after LPS injection animals were treated with L-N^*ω*^-Nitroarginine (L-NNA; 0.5 mg.kg^−1^ IV; 0.2 ml; Sigma-Aldrich, St. Louis, MO, USA) and then fluid resuscitated with normal saline (40 ml.kg^−1^ in 30 minutes), and one hour after fluid resuscitation, animals were treated with L-Arginine hydrochloride (100 mg.kg^−1^ IV; 0.2 ml; Sigma-Aldrich, St. Louis, MO, USA).

**Figure 1:**
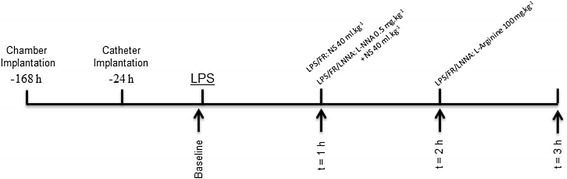
**Schematic representation of the experimental protocol.** After baseline determination of hemodynamic and microcirculatory parameters and evaluation of leukocyte-endothelial interactions (Baseline) LPS was administrated. Sequential measurements were performed after one (t = 1 h), two (t = 2 h), and three (t = 3 h) hours of LPS injection (arrows). Blood sampling for biochemical and hematological analysis was performed in all groups at the end of the study period (t = 3 h). LPS/FR, endotoxemic and fluid resuscitated animals; LPS/FR/LNNA, endotoxemic, fluid resuscitated, and L-NNA + L-Arginine treated animals; NS, normal saline.

As shown in Figure 1, sequential measurements of hemodynamic and microcirculatory parameters and evaluations of leukocyte-endothelial interactions were performed in four time points: at baseline (Baseline) and after one (t = 1 h), two (t = 2 h), and three (t = 3 h) hours of LPS injection. At t = 1 h and t = 2 h, these measurements were performed immediately before fluid or drug administration. Blood sampling for biochemical and hematological analysis was performed in all groups at the end of the study period (t = 3 h).

### Survival analysis

After the intravital microscopy phase of the experiments, animals were returned to their individual cage in the *vivarium* with free access to water and standard chow and monitored for survival three times per day for 7 days. After 7 days, surviving animals were euthanized by a lethal dose of pentobarbital.

### Statistical analysis

Results are expressed as means ± standard deviation of the mean (SD), unless otherwise noted. Microvascular diameters and FCD data are presented as ratios relative to baseline values. All hemodynamic and microcirculatory measurements were compared to baseline of the same group and between groups at the same time point. Statistical differences within and between groups were determined by Friedman and Kruskal-Wallis tests, followed, when appropriate, by Dunn’s multiple-comparison test for *post hoc* analysis. Survival curves were obtained using Kaplan-Meier procedure and Mantel-Cox log-rank test was applied for determination of significant differences between study groups. All statistical analyses were performed using GraphPad Prism 6.03 (GraphPad Software, La Jolla, CA, USA) and the significance level was set as *p* <0.05.

## Results

The average body weight of the hamsters was 75.6 ± 6.8 g with no significant differences among groups. All animals survived the intravital microscopy phase of the experimental protocol and underwent survival analysis.

### Hemodynamic alterations

MAP and HR basal values were comparable to control values from healthy animals reported in the literature [17] and no significant differences among the experimental groups were found. Systemic administration of LPS elicited similar reductions of MAP in all groups (Table 1; t = 1 h). At t = 2 h, MAP was significantly higher in LPS/FR/LNNA group than in LPS one. At t = 3 h, MAP was significantly higher in LPS/FR/LNNA group than in both LPS and LPS/FR groups (Table 1). HR was comparable among groups at each time point (Table 1).

**Table 1:** **Mean arterial pressure and heart rate evolution during the experimental period**

		**LPS**	**LPS/FR**	**LPS/FR/LNNA**
Mean arterial pressure (mmHg)	Baseline	110.8 ± 7.7	104.9 ± 5.0	115.3 ± 10.4
	t = 1 h	80.3 ± 10.6^†^	81.4 ± 5.4^†^	87.0 ± 11.4†
	t = 2 h	70.3 ± 9.7^†^	80.6 ± 7.9^†^	107.0 ± 18.9*
	t = 3 h	70.8 ± 7.5^†^	75.7 ± 9.2^†^	100.2 ± 14.3*^§^
Heart rate (bpm)	Baseline	430.0 ± 25.8	423.6 ± 31.0	417.4 ± 41.0
	t = 1 h	425.7 ± 39.9	419.2 ± 32.7	440.5 ± 51.4
	t = 2 h	410.2 ± 50.3	438.9 ± 33.1	395.6 ± 48.2
	t = 3 h	392.7 ± 49.3^†^	419.1 ± 29.5	426.2 ± 77.8

Data are presented as means ± SD for each group. LPS, endotoxemic animals (n = 10); LPS/FR, endotoxemic and fluid resuscitated animals (n = 10); LPS/FR/LNNA, endotoxemic, fluid resuscitated, and L-NNA + L-Arginine treated animals (n = 10). ^†^p <0.05 vs. group baseline; *p <0.05 vs. LPS group at the same time point; ^§^p <0.05 vs. LPS/FR group at the same time point.

### Leukometry and evaluation of leukocyte-endothelial interactions

**Figure 2:**
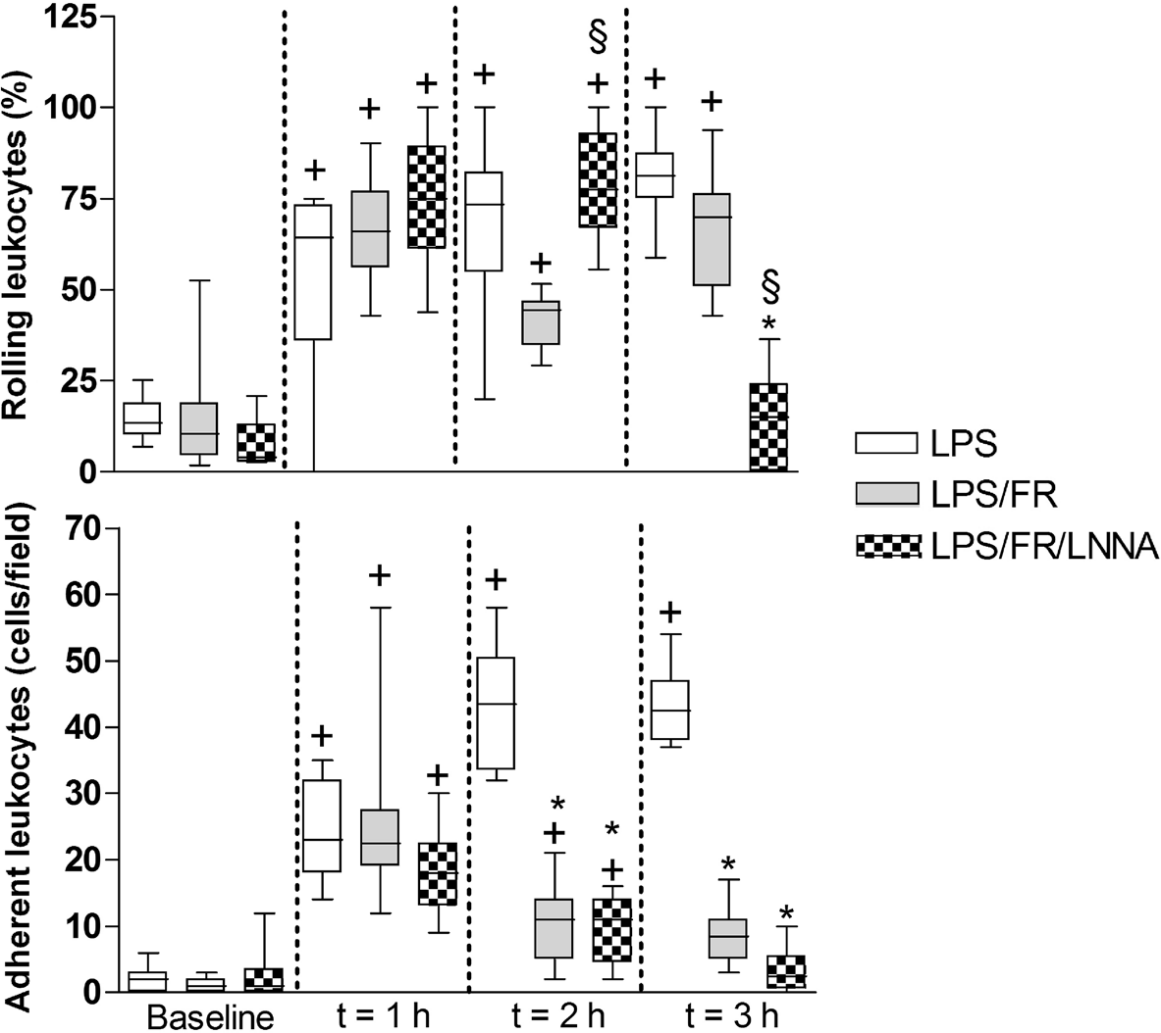
**Sequential evaluation of leukocyte-endothelial interactions.** The number of rolling leukocytes is expressed as percentage of the number of passing leukocytes. The number of adherent leukocytes is expressed as the number of adherent cells per field. Sequential measurements were performed at baseline and after one (t = 1 h), two (t = 2 h), and three (t = 3 h) hours of LPS injection. LPS, endotoxemic animals (n = 10); LPS/FR, endotoxemic and fluid resuscitated animals (n = 10); LPS/FR/LNNA, endotoxemic, fluid resuscitated, and L-NNA + L-Arginine treated animals (n = 10). ^†^ *p* <0.05 *vs.* group baseline; *p <0.05 *vs.* LPS group at the same time point; ^§^ *p* <0.05 *vs.* LPS/FR group at the same time point.

**Figure 3:**
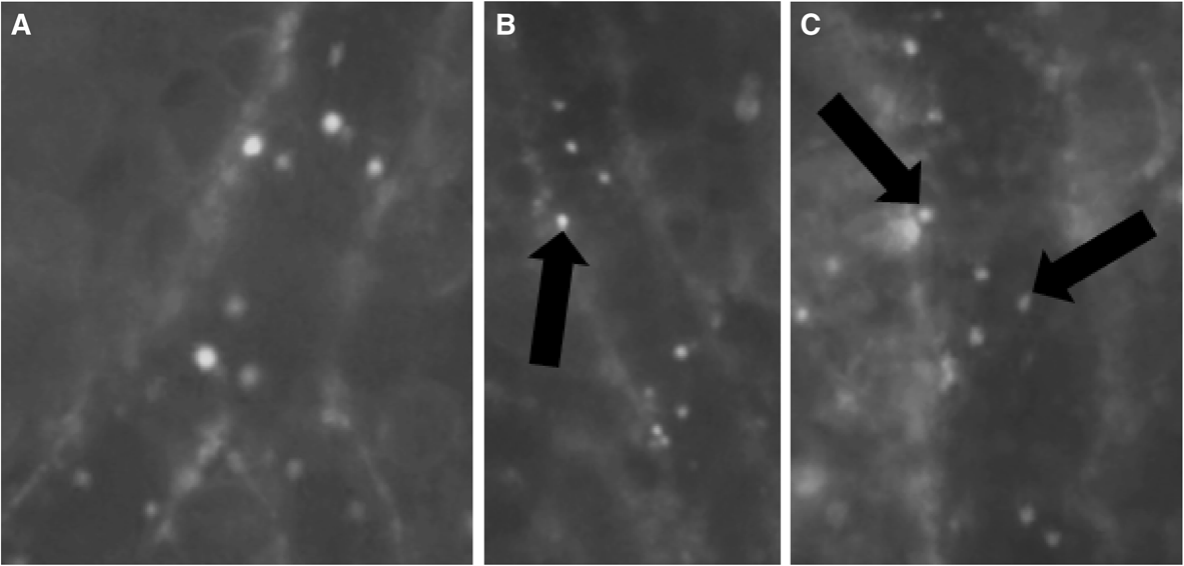
**Representative photomicrographs of the skinfold microcirculation after** ***in vivo*** **staining of leukocytes with rhodamine 6G (t = 3 h).** Almost all leukocytes observed in **(A)** are adhered to the venular endothelium. Adhered leukocytes are marked by black arrows in **(B)** and **(C)**. **(A)** LPS group; **(B)** LPS/FR group; **(C)** LPS/FR/LNNA group. Final magnification: 800x.

Leukometry decreased after the induction of endotoxemia (comparing to control values from healthy animals reported in the literature [17]). The LPS/FR group had significantly higher white blood cell counts compared to LPS group (Table 2). Injection of LPS significantly increased the percentage of rolling leukocytes in all groups (Figure 2; t = 1 h). Fluid resuscitation reduced this percentage (compared with LPS group) while L-NNA treatment inhibited the fluid response (*p* <0.05 for LPS/FR/LNNA *vs.* LPS/FR group) (Figure 2; t = 2 h). L-Arginine administration significantly reduced the percentage of rolling leukocytes at t = 3 h (*p* <0.05 for LPS/FR/LNNA *vs.* LPS and LPS/FR groups) (Figure 2; t = 3 h). Endotoxemia significantly increased the number of adhered leukocytes in all groups. At t = 2 h and t = 3 h, fluid resuscitation decreased leukocyte adherence regardless of L-NNA or L-Arginine treatment (Figure 2). Representative photomicrographs are shown in Figure 3.

**Table 2:** **Biochemical and Hematological Parameters**

	**LPS**	**LPS/FR**	**LPS/FR/LNNA**
Arterial lactate (mmol.l^−1^)	7.92 ± 1.30	3.42 ± 0.44*	3.56 ± 0.73*
pH	7.25 ± 0.04	7.33 ± 0.03*	7.18 ± 0.07*^§^
Bicarbonate (mmol.l^−1^)	21.50 ± 1.00	23.25 ± 0.95	22.95 ± 3.30
Base excess (mmol.l^−1^)	−4.7 ± 3.3	−2.1 ± 0.9*	−4.4 ± 0.3^§^
Hematocrit (%)	55.75 ± 1.70	50.00 ± 2.70*	47.70 ± 1.33*
Blood Glucose (mg.dl^−1^)	32.00 ± 9.20	45.00 ± 11.22*	38.20 ± 7.48
Leukometry (n/mm^3^)	1,553 ± 493	3,305 ± 675*	2,330 ± 457

Blood sampling for biochemical and hematological analysis was performed in all groups at the end of the study period (t = 3 h). Data are presented as means ± SD for each group. LPS, endotoxemic animals (n = 10); LPS/FR, endotoxemic and fluid resuscitated animals (n = 10); LPS/FR/LNNA, endotoxemic, fluid resuscitated, and L-NNA + L-Arginine treated animals (n = 10). *p <0.05 vs. LPS group; ^§^p <0.05 vs. LPS/FR group.

### Arteriolar and venular mean internal diameters and arteriolar blood flow score

**Figure 4:**
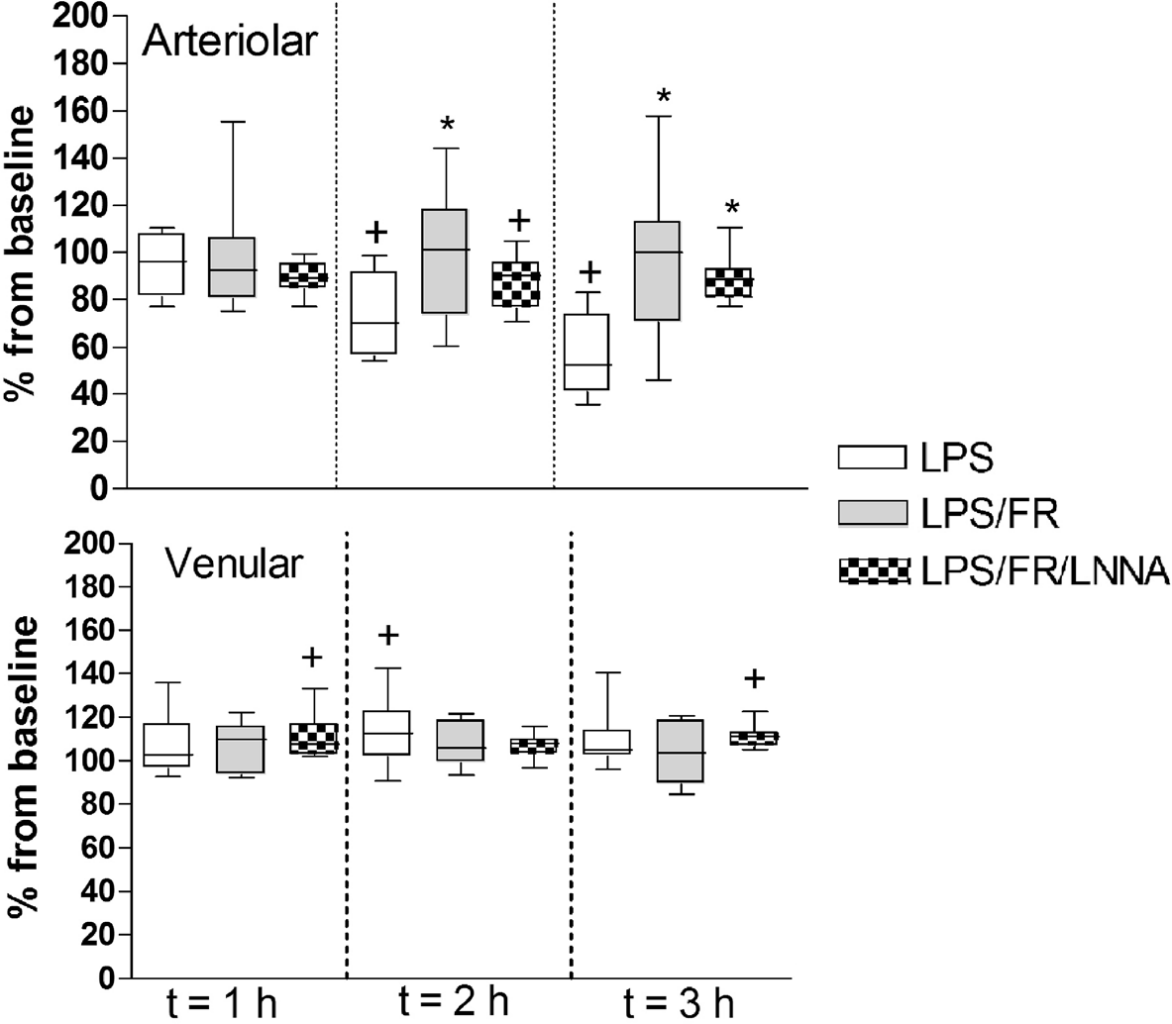
**Evolution of arteriolar and venular mean internal diameters during the experimental period.** Values are presented as ratios relative to baseline values. Sequential measurements were performed after one (t = 1 h), two (t = 2 h), and three (t = 3 h) hours of LPS injection. LPS, endotoxemic animals (n = 10); LPS/FR, endotoxemic and fluid resuscitated animals (n = 10); LPS/FR/LNNA, endotoxemic, fluid resuscitated, and L-NNA + L-Arginine treated animals (n = 10). ^†^ *p* <0.05 *vs.* group baseline; *p <0.05 *vs.* LPS group at the same time point.

**Figure 5:**
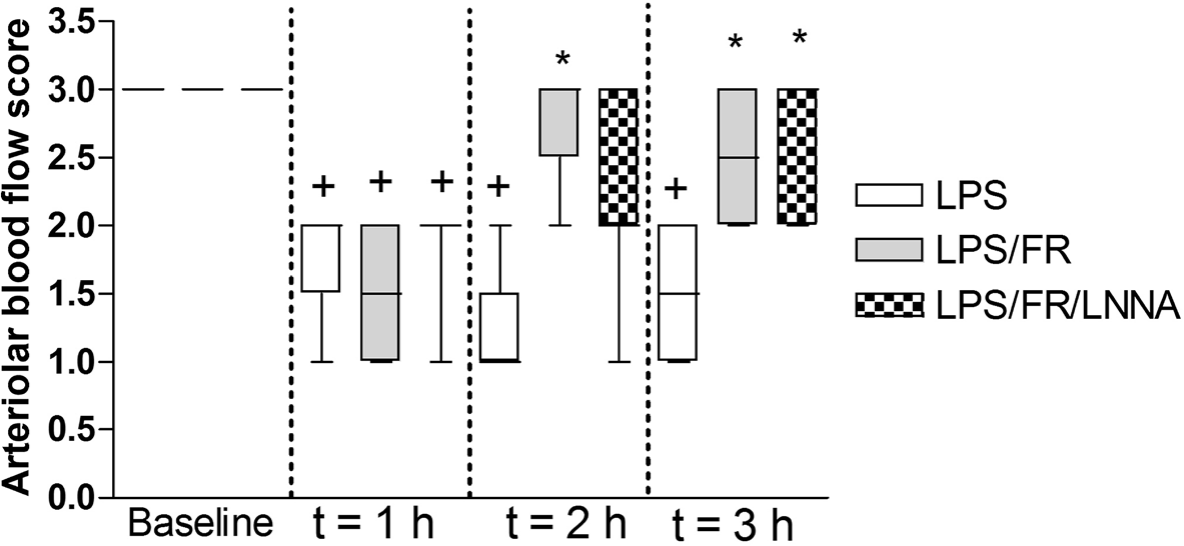
**Evolution of arteriolar blood flow score during the experimental period.** 0, no flow; 1, intermittent flow; 2, sluggish flow; 3, normal flow. Sequential measurements were performed at baseline and after one (t = 1 h), two (t = 2 h), and three (t = 3 h) hours of LPS injection. LPS, endotoxemic animals (n = 10); LPS/FR, endotoxemic and fluid resuscitated animals (n = 10); LPS/FR/LNNA, endotoxemic, fluid resuscitated, and L-NNA + L-Arginine treated animals (n = 10). ^†^ *p* <0.05 *vs.* group baseline; *p <0.05 *vs.* LPS group at the same time point.

At baseline, there was no significant difference in arteriolar and venular mean internal diameters or in arteriolar blood flow score between groups. Endotoxemia significantly reduced arteriolar mean internal diameter in the LPS group at t = 2 h and t = 3 h when compared to baseline. At t = 2 h, arteriolar mean internal diameter was significantly smaller in the LPS group compared with the LPS/FR one. At this same time point, there was no significant difference between LPS/FR/LNNA group and both LPS and LPS/FR groups. At t = 3 h, arteriolar mean internal diameter was significantly smaller in the LPS group than in both LPS/FR and LPS/FR/LNNA ones (Figure 4). Arteriolar blood flow score decreased in all groups after LPS administration. Fluid resuscitation attenuated this reduction independently of L-NNA or L-Arginine treatments (Figure 5). In terms of venular mean internal diameter, LPS elicited venular vasodilatation but there was no significant difference between groups at any time point (Figure 4).

### Capillary perfusion (FCD and RBC-Vel)

**Figure 6:**
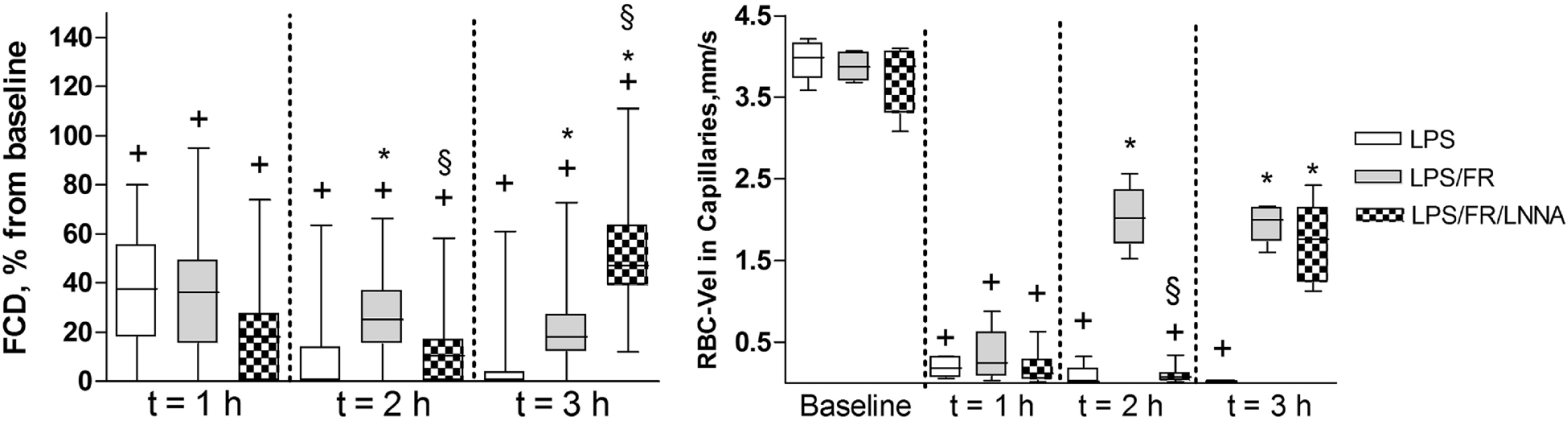
**Evolution of functional capillary density (FCD) and red blood cell velocity in capillaries (RBC-Vel) during the experimental period.** Values of FCD are presented as ratios relative to baseline values. Sequential measurements were performed at baseline and after one (t = 1 h), two (t = 2 h), and three (t = 3 h) hours of LPS injection. LPS, endotoxemic animals (n = 10); LPS/FR, endotoxemic and fluid resuscitated animals (n = 10); LPS/FR/LNNA, endotoxemic, fluid resuscitated, and L-NNA + L-Arginine treated animals (n = 10). ^†^ *p* <0.05 *vs.* group baseline; *p <0.05 *vs.* LPS group at the same time point; ^§^ *p* <0.05 *vs.* LPS/FR group at the same time point.

At baseline, FCD and RBC-Vel did not significantly differ between studied groups and LPS administration markedly decreased FCD and RBC-Vel. In the LPS/FR group, fluid resuscitation significantly attenuated the fall of capillary perfusion parameters at t = 2 h and t = 3 h. In the LPS/FR/LNNA group, L-NNA treatment inhibited this response at t = 2 h while L-Arginine administration significantly increased FCD and RBC-Vel at t = 3 h (Figure 6).

### Biochemical and hematological parameters

Biochemical and hematological parameters are presented on Table 2. Arterial lactate concentration was significantly higher in LPS group compared to the other groups, but there was no statistical difference between LPS/FR and LPS/FR/LNNA groups. Arterial pH and BE were lower in LPS group compared with LPS/FR one. Arterial pH was even lower in LPS/FR/LNNA group. Blood glucose was significantly lower in LPS group compared to LPS/FR one. Hematocrit was significantly higher in LPS group compared to other groups, but there was no statistical difference between LPS/FR and LPS/FR/LNNA groups.

### Seven days survival

**Figure 7:**
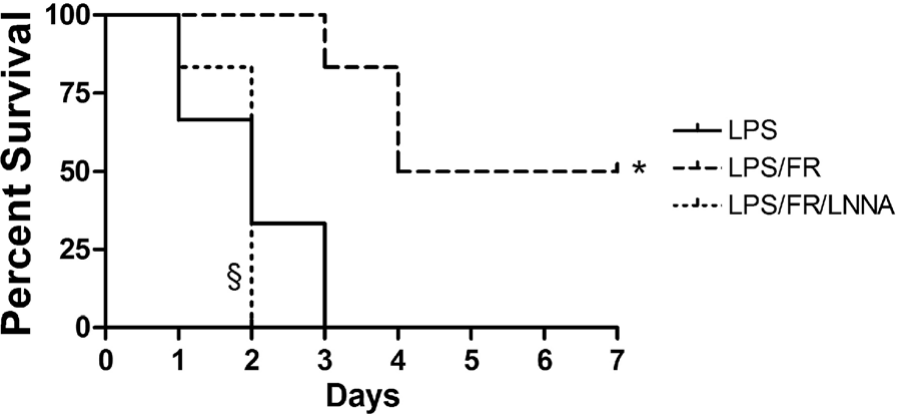
**Kaplan-Meier survival curves.** LPS, endotoxemic animals (n = 10); LPS/FR, endotoxemic and fluid resuscitated animals (n = 10); LPS/FR/LNNA, endotoxemic, fluid resuscitated, and L-NNA + L-Arginine treated animals (n = 10). *p <0.05 *vs.* LPS group; ^§^ *p* <0.05 *vs.* LPS/FR group.

Median survival time was 2, 2, and 5.5 days for LPS, LPS/FR/LNNA, and LPS/FR groups, respectively (*p* <0.05 for LPS/FR *vs.* LPS; *p* <0.05 for LPS/FR *vs.* LPS/FR/LNNA; Figure 7).

## Discussion

LPS-induced endotoxemia in rodents is a well-established experimental model that reproduces many of clinical features of sepsis syndrome. Although some authors do not consider it a model of sepsis, but rather a model of endotoxemia, it is commonly used in the study of the pathophysiology and treatment of sepsis and septic shock [17-19]. Endotoxin administration offers advantages over other models of sepsis, since experiments are easy to perform and the dose can be controlled and adjusted to the actual body weight of the animal. Another advantage of this model is that homogeneity and reproducibility of microvascular responses are consistently obtained [17].

The hamster skinfold window chamber model developed by Endrich and co-workers [14] is widely used for microvascular studies in unanesthetized animals [20-25]. Unlike acute microcirculatory models, this one allows a recovery period between surgical manipulation for chamber implantation and experiments. This period is crucial for the recovery of microcirculatory function affected by surgical trauma. Furthermore, compared to other microcirculatory models, skinfold chamber model experiments can be performed without induction of general anesthesia, which has hemodynamic, immune, and microvascular effects of its own. It has already been shown that microcirculatory changes in the window chamber model during shock correlate to changes in vital organs [20]. Hamsters tolerate the chamber and catheters well and show no signs of discomfort. Three different tissues are present in the chamber: skeletal muscle, adipose tissue, and subcutaneous connective tissue. Even using a chronic microcirculatory model, such as the skinfold window chamber, *in vivo* observations of the microcirculation cannot be extended over several days because the tissues present in the chamber evolve according to the pathology to which they are subjected and to the wound healing process that occurs as a consequence of long term tissue exposure to chamber environment. Thus, the longitudinal data obtained would not be reliable.

The endotoxin dose used in our study was adjusted to affect microcirculatory parameters without induction of severe hypotension. The normal saline volume administered for fluid resuscitation (40 ml.kg^−1^ in 30 minutes) was chosen based on previously published experimental study which showed that this volume was able to improve microcirculatory parameters in endotoxemic hamsters increasing survival rate [6]. Several L-NNA doses were tested on a pilot study in order to find the one with lesser systemic repercussions, which was chosen for the present investigation. The same process was followed for L-Arginine dose selection.

L-NNA is one of the first synthetic nitric oxide synthase (NOS) inhibitors that interacts with all NOS noncovalently. Its coupling with NOS-2 is immediate and rapidly reversible with arginine supplementation. However, the binding to NOS-1 and NOS-3 is time-dependent with relatively slow reversal [26]. L-NNA shows low toxicity and has been extensively used in experimental and clinical studies mostly addressing sepsis. In these studies, L-NNA is normally used to modulate hemodynamics in septic conditions resulting in reversal of sepsis-associated hypotension and increased systemic vascular resistance. Besides these beneficial effects, NOS inhibition is associated with decreased cardiac output, lowered regional blood flow, and increased mortality. L-NNA effects are reversed by L-Arginine infusions [27].

L-Arginine is the physiological substrate of NO synthesis in a process involving NOS. Its IV infusion is able to improve NO bioavailability and endothelial function in septic patients when used as an adjunctive treatment [28]. When used as monotherapy, parenteral L-Arginine was associated with deleterious effects to animals in septic shock [29]. As the precursor of NO, L-Arginine evokes dose and endothelial-dependent arteriolar dilatation [30]. Thus, major side effects are related to induction of vasodilatation and hypotension [31]. Nevertheless at the dose and infusion rate used in our study, L-Arginine was safe and caused no macro-hemodynamic effects.

Since endotoxemia is a known up-regulator of leukocyte-endothelial interactions and because the rise in these interactions is related to microcirculatory impairment [6], we have performed intravital fluorescence microscopy to investigate leukocyte kinetics. Leukocyte migration occurs in a step-wise process: first leukocytes roll along the vascular endothelium, then they firmly adhere to it and finally emigrate from the blood to inflamed tissues. Thus, quantitation of rolling and adherent leukocytes may reasonably reflect the extent of the inflammatory response. In our study, fluid resuscitation has prevented leukocyte rolling by modulation of NO synthesis since this effect was abolished after NOS inhibition with L-NNA and markedly restored after L-Arginine administration. NO is a potent *in vivo* inhibitor of leukocyte-endothelial interactions, although the mechanisms involved on this suppression are not completely understood [32,33]. We were unable to demonstrate a similar behavior regarding leukocyte adhesion as both fluid resuscitated groups (LPS/FR and LPS/FR/LNNA) presented significant and maintained reduction of leukocyte adherence. This result might be related to different adhesion molecules involved; while rolling involves the selectin family of adhesion molecules, firm adhesion involves β_2_-integrins (expressed in leukocytes) and the endothelial intercellular adhesion molecule-1 (ICAM-1) [34,35]. NO modulatory activity on selectin expression has already been demonstrated supporting our findings [33,36,37]. Considering that NO capacity to inhibit ICAM-1 expression in LPS-stimulated endothelial cells has also been demonstrated [32], our results suggest that fluid resuscitation effects on leukocyte adhesion are not solely related to the NO pathway and other mechanisms might be involved. So, the discrepancy between our observations could be attributed to complex and overlapping cellular interactions found in *in vivo* studies.

The observed leukometry difference after endotoxemia induction is likely to be related to activation and emigration of neutrophils out of the microvascular bed and to an accumulation of leukocytes in organs such as lung and liver (leukocyte sequestration). This leukopenia becomes obvious 30 minutes after LPS infusion and usually persists for 8 hours after a single dose [17]. In our study, fluid resuscitated animals in LPS/FR group showed greater leukocyte count. This protective effect was blunted by NOS inhibition suggesting NO participation on its mechanism, probably by inhibition of leukocyte-endothelial interactions. Considering that migration is a one-way process that starts early after endotoxemia induction, it is understandable that L-NNA consequences may not be reversed by later L-Arginine administration.

Once migration has occurred, tissue damage happens as consequence of the release of an array of inflammatory mediators, cytotoxic enzymes, and oxygen radicals. So, generalized activation and sequestration of neutrophils is likely to contribute to the widespread microvascular injury and subsequent endothelial damage observed in sepsis, which represents a central step in development and progression of multiple organ failure [13,24]. Since tissue and organ accumulation of leukocytes is triggered and amplified by pro-inflammatory mediators, it may be suggested that resultant leukopenia could be a marker of the inflammatory process that leads to microvascular dysfunction. In this way, the observed anti-inflammatory effects of fluid resuscitation could be beneficial to the microcirculation. Considering FCD and RBC-Vel temporal evolution, our study showed that fluid resuscitation was associated with significant attenuation of capillary perfusion deficits induced by LPS administration. Furthermore, we could observe that previous NOS inhibition with L-NNA blunted this response suggesting that, at least in part, the beneficial effects of fluid resuscitation on microcirculation are also linked to NO pathway. The improved capillary perfusion observed after administration of L-Arginine favors this hypothesis.

Fluid resuscitation prevented arteriolar vasoconstriction in LPS/FR group. It is possible that fluid-induced improvements of arteriolar blood flow velocity (as compared with LPS group) may have dilated arterioles by a mechanism dependent on shear stress. Endothelial cells respond to physical forces induced by blood flow (shear stress) with upregulation of production, release, and/or activity of factors linked to vasodilatation, such as the endothelial NOS [38,39]. Furthermore blood flow-associated mechanical alterations of the endothelium result in attenuated sympathetic outflow [40]. In conjunction with the vasodilator stimuli, this could explain why MAP did not increase after fluid resuscitation in this group. L-NNA administration inhibited this effect: unlike what was seen in LPS/FR group, despite an improvement of arteriolar blood flow velocity, in LPS/FR/LNNA group fluid resuscitation was associated with increased MAP and arteriolar vasoconstriction. L-arginine administration partially reversed the effects of NOS inhibition.

Since capillary perfusion differences between LPS/FR and LPS/FR/LNNA groups cannot be entirely explained by changes in arteriolar mean internal diameter or by macro-hemodynamic changes, we may also speculate that anti-inflammatory and antithrombotic activities of NO have been crucial to capillary perfusion differences observed between groups [41,42]. Capillary obstruction by microthrombi or leukocyte plugs and an increased presence of rolling or adherent leukocytes in venules may hamper adequate capillary flow [22,43]. In fact, as previously stated, we could observe significant differences on leukocyte rolling among groups that may have contributed to observed capillary perfusion results. Although the occurrence of vascular microthrombosis has not been evaluated in our study, some authors have already shown that L-Arginine is able to inhibit platelet aggregation in healthy and ill subjects [44,45].

Lower values of pH, HCO_3_, and BE found in LPS group compared with LPS/FR one are indicative of metabolic acidosis, probably secondary to tissue hypoperfusion. In this group, the presence of metabolic acidosis associated with hyperlactatemia denotes a high risk clinical condition [46,47]. On the other hand, lower arterial lactate concentrations observed in fluid resuscitated groups suggest that these groups had, at the moment of blood sampling, better tissue perfusion compared with the LPS group corroborating our microcirculatory findings. In a similar way, a correlation between tissue perfusion and microvascular functional recovery has already been reported by Hangai-Hoger et al. [48]. L-Arginine infusion may be responsible for the decreased pH and BE observed in the LPS/FR/LNNA group. This change has already been described in the literature and it is likely due to hydrogen and chloride ions present on L-Arginine hydrochloride solutions [49].

The observed development of hypoglycemia after LPS administration is a common finding during inflammatory states in small animals [50]. Thus, the relative higher glucose level found in LPS/FR group may be related to lower inflammatory response. Unfortunately, assessment of the involvement of NO pathway on this difference was hindered by complex actions of L-NNA and L-Arginine on glycemia [49,51].

The improved arteriolar blood flow and capillary perfusion found in LPS/FR and LPS/FR/LNNA groups were translated into increased survival only in the LPS/FR group. This could be intriguing as we have already demonstrated that improved microcirculatory parameters are correlated with improved survival rates [6]. Some hypotheses can be deemed in this respect. First, there is a huge difference between L-Arginine and L-NNA half-lives; IV L-Arginine is short-lived (40–60 minutes half-life) while L-NNA’s half-life in septic organisms can reach 23 hours [31,52,53]. Taking this into account, it is perceived that the beneficial actions of L-Arginine are relatively transient compared to the longer lasting detrimental effects of the NOS inhibitor. Thus, it can be speculated that the microcirculatory improvement observed in LPS/FR/LNNA group at t = 3 h would not have been sustained. Secondly, and complementing the first hypothesis, NOS inhibition by L-NNA occurs in such a way that replacement with L-Arginine reverses more easily the inhibition of the inducible isoform [26]. As the concentration of L-Arginine decays, the constitutive isoforms (NOS-1 and NOS-3) are readily reinhibited by L-NNA while NOS-2 activity can last longer. Of note, this profile of NOS inhibition is just the opposite of that desired during sepsis, since specific reduction of NOS-2 activity has proved beneficial in reducing mortality in experimental models of sepsis, as it can reduce unwanted cardiovascular and perfusional consequences of increased NO synthesis, while preserving other important NO actions, such as host defense [54-57]. Finally, both activation (L-Arginine) and inhibition (NOS inhibitors) of NO synthesis have been associated with increased macromolecular extravasation in the microcirculation [58]. Moreover, increased capillary leakage has been correlated with increased mortality [59,60]. These findings are in consonance with survival studies using small animals that have consistently not favored the use L-Arginine or NOS inhibitors during sepsis [27,61]. Therefore, the use of these drugs in LPS/FR/LNNA group may have independently contributed for the lack of any survival benefit. Our hematocrit results did not corroborate the altered capillary leakage hypothesis. This hematological parameter has been used as an indirect marker of plasma leakage in the context of endothelial-damaging infectious diseases like dengue virus infection [62]. Hematocrit increased after endotoxemia induction in LPS group (comparing to control values from healthy animals reported by the literature [23]), suggesting increased capillary leakage. The same response was not observed in fluid resuscitated groups. The short interval between treatments (L-NNA and L-Arginine) and blood sampling may have biased these results. On the other hand, we cannot exclude that fluid-induced anti-inflammatory effects may have influenced capillary leakage. For technical or protocol reasons, it was not possible to prolong *in vivo* observation of the microcirculation, perform a direct assessment of capillary leakage, or proceed with sequential blood sampling, limiting the validation of these hypotheses.

Taking all our results, we may suggest a possible explanation for the contradictory effects of fluid resuscitation on different trials, sometimes favoring survival and other times mortality. Pathogens and patients specificities may determine heterogeneous endothelium dysfunction. Patients with systemic chronic comorbidities, long lasting diseases, or malnourished ones may have alterations of the endothelial function (such as decreased NO syntheses); in a similar way, some pathogens such as dengue virus and *Plasmodium* are related to significant endothelium damage [12,49,62,63]. On these cases, impaired NO-dependent endothelial functions could preclude the beneficial effects of fluid resuscitation and even enhance its adverse effects, such as tissue edema [58]. Excessive interstitial edema may contribute to dysfunction of some organs like lungs (acute respiratory distress syndrome), brain (encephalopathy), and intestines (intra-abdominal hypertension syndrome) playing an important role on progression of sepsis syndrome till death. Our “endothelial/NO-centered” hypothesis cannot be completely proven by our data. Thus, the contradictions with respect to fluid therapy in sepsis still need further research to be better clarified/resolved.

Nitric oxide involvement in the response to fluid resuscitation has already been described and studied in models involving isolated organs and tissues and *in vivo* for changes in macro-hemodynamic parameters and/or in surrogate markers of tissue perfusion [10]. However, in our study, we have demonstrated effects of nitric oxide using an experimental model that allows direct observation of the microcirculation. Thus, it was possible to directly observe microcirculatory vasomotor phenomena and capillary perfusion. The experimental model used also allowed direct assessment of leukocyte-endothelial interactions providing important data about effects of nitric oxide on these interactions. Furthermore, as previously stated, the skinfold chamber model permits reliable microcirculatory studies without the influence of surgical trauma or general anesthesia on microcirculatory function. Considering that microcirculatory alterations are more associated with patient survival in sepsis than macro-hemodynamic changes and that *in vivo* observations are more translational than observations of isolated organs and tissues, we believe that our study adds new and important information to current knowledge on the subject [6].

We are aware that our study has some limitations. Initially, we recognize that observation of the skin and subcutaneous muscle microcirculation may not be representative of microcirculatory changes in splanchnic organs. Given the crucial importance of splanchnic perfusion in the pathophysiology of sepsis, this could be considered a limitation of the skinfold window chamber model. However, the first reactions after endotoxin administration seem to be comparable in different organs [17]. In the second place, comparing fluid resuscitated to unresuscitated animals may sound worthless but it fulfills the main objectives of the study. Although we know that fluid resuscitation is recommended in early management of severe sepsis and septic shock, our control animals (LPS group) were not fluid resuscitated because this study was designed to evaluate mechanisms behind microcirculatory effects of fluid therapy which demands an untreated group. Thirdly, animals were fluid resuscitated with normal saline, a chloride rich crystalloid solution. Despite the observed microcirculatory benefits, recent findings suggest that hyperchloremic resuscitation may be associated with worse outcomes, particularly in patients with septic shock [64,65]. Since we did not intend to compare different choices of resuscitation fluids, we may only suggest that fluid resuscitation with normal saline is better than no resuscitation. Future investigations could focus on effects of different fluid regimens. In fourth place, in our study, NOS was acutely inhibited by L-NNA administration. This may not completely mimic mechanisms involved on prolonged endothelial dysfunction observed during sepsis. It is already known that the cardiovascular system adapts to an acute inhibition of NO synthesis in a different manner than observed with its chronic lack [66]. A similar process may be found in the microcirculation in which endothelial cells of skeletal muscle arterioles adapt to chronic lack of NO and maintain dilator responses to flow/shear stress by up-regulating the synthesis of other gender-dependent dilatatory mediator [67-70]. Thus, endothelial response to NOS inhibitors is dependent on duration of use of such inhibitors and our results could have been different if chronically L-NNA treated animals were used. Finally, we did not directly measure NO derivatives (nitrate/nitrite; NOx) plasma levels, instead we have performed inhibitory and stimulatory functional tests of NO pathway participation on fluid-induced alterations, applying a validated experimental design [10]. This must be taken in account on the interpretation of our results and also the fact that we have used L-NNA, a nonselective NOS inhibitor. Some of our results could be different if we had used more selective NO inhibitors, such as 1400 W.

## Conclusions

In the present study, LPS-induced endotoxemia resulted in severe dysfunction of hamster skinfold microcirculation, which was greatly reduced by fluid resuscitation therapy. The fluid therapy improved capillary perfusion deficit and attenuated the inflammatory response, as demonstrated by decreased leukocyte-endothelium interactions and sequestration. Importantly, the improvement of skinfold microcirculation corresponded with longer survival of the animals emphasizing the translational aspect of findings. The underlying mechanism of fluid therapy is likely to be the restoration of nitric oxide bioavailability, because inhibition of NOS prevented many of the beneficial effects of fluid therapy. Nevertheless, further investigations are required to confirm these results in experimental models that are closer to conditions of human sepsis.

## Abbreviations

NO: Nitric oxide
MAP: Mean arterial blood pressure
HR: Heart rate
FCD: Functional capillary density
RBC-Vel: Red blood cell velocity in capillaries
IV: Intravenously
HCO3: Bicarbonate level
BE: Base excess
LPS: Escherichia coli serotype 055,B5 lipopolysaccharide
L-NNA: L-N*ω*-Nitroarginine
NOS: Nitric oxide synthase
